# Pigmented, Birefringent Material Causing Nasolacrimal Duct Obstruction

**DOI:** 10.1007/s12070-025-05526-0

**Published:** 2025-06-03

**Authors:** Angela L. Xu, César A. Briceño, Vivian Lee

**Affiliations:** 1 Perelman School of Medicine, University of Pennsylvania, Philadelphia, USA; 2 Scheie Eye Institute, Department of Ophthalmology, Perelman School of Medicine, University of Pennsylvania, Philadelphia, USA

**Keywords:** Epiphora, Granulomatous inflammation, Cosmetics, Nasolacrimal duct obstruction

## Abstract

We present a case of a woman with epiphora secondary to complete nasolacrimal duct obstruction due to granulomatous inflammation around pigmented, birefringent foreign bodies, consistent with cosmetics. History is critical when evaluating epiphora to differentiate an innate melanocytic process, such as melanoma, from exogenous sources such as cosmetics on histopathology.

## Introduction

Mascara and pigmented makeups are widely used and can cause various conjunctival and punctal pathologies. A patient with epiphora, believed to be secondary to dry eye, was found instead to have complete nasolacrimal duct obstruction (NLDO) from granulomatous inflammation around pigmented, birefringent material.

## Case Report

A 35-year-old woman with a history of allergic conjunctivitis, dry eyes, and chronic sinusitis was referred to clinic for a second opinion on excessive tearing of her left eye. For the last two years she struggled with a unilateral weeping sensation and early morning eyelid crusting. Her symptoms were unabated by frequent warm compresses, lid scrubs, and artificial tears. She used daily contact lenses, for which she was compliant with routine care.

During her first visit, she had 20/20 vision bilaterally with normal pressures. Slit-lamp exam was notable for bilateral 2 + meibomian gland dysfunction, conjunctivochalasis, punctate epithelial erosions, and a fast tear-breakup time. A Jones 1 dye test was positive and patent puncta were visualized during this visit. A 1-week contact lens break and nighttime erythromycin ointment was prescribed. Upon her return to clinic, her exam was improved with decreased erosions and staining, but there was no symptomatic improvement. Given her exam and previously positive Jones test, there was low concern for an occluded tear duct and irrigation was deferred. However, due to the unilateral nature of her symptoms, referrals to both oculoplastic and dry eye specialists were given.

At her dry eye appointment 6 months later, erythromycin ointment as well as a trial of tobramycin/dexamethasone eye drops, was started. Neither of these interventions improved her symptoms. Four months later, she presented to oculoplastics clinic where exam showed clear discharge from her left eye when pressing on the ipsilateral lacrimal sac. Probing and irrigation showed complete NLDO of the left eye with patent canaliculi. She was scheduled to undergo external dacryocystorhinostomy. A standard approach to dacryocystorhinostomy was pursued and a lacrimal sac biopsy was submitted to surgical pathology. The patient returned to clinic 2 weeks later and was doing well post-operatively with marked improvement in her symptoms.

**Fig. 1:**
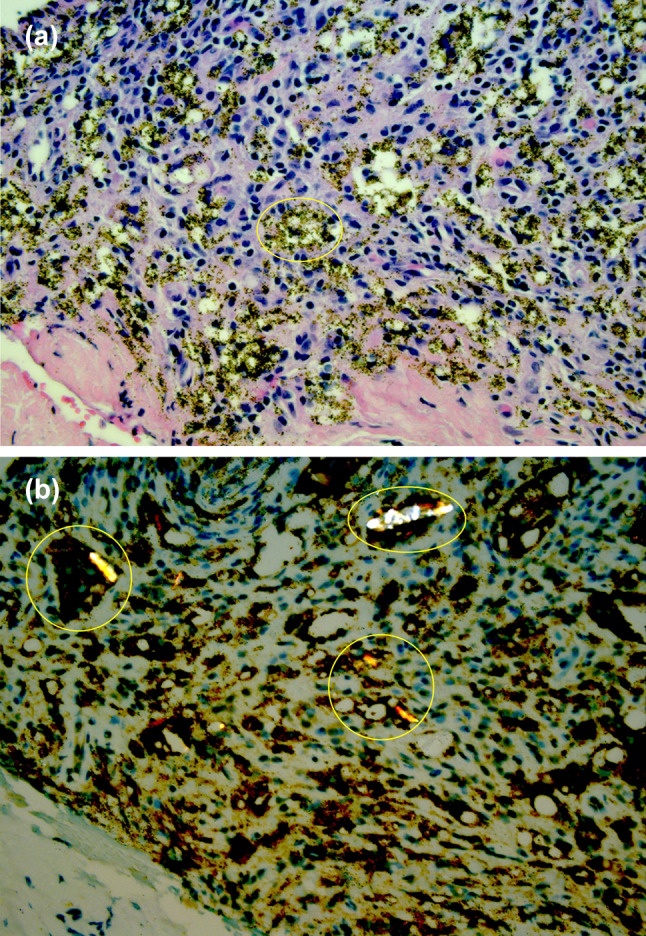
**A** Photomicrograph of nasolacrimal duct mucosa demonstrating granulomatous inflammation with associated pigmented granules (example area indicated by yellow circle). **B** Macrophages engulfing polarizable foreign bodies (yellow circles) (Hematoxylin–eosin and CD68 staining respectively, both at 100× magnification)

Histopathology of the lacrimal sac biopsy revealed soft tissue with focal areas of granulomatous inflammation, confirmed by CD68 and CD163 staining. These areas were associated with pigmented granules and polarizable foreign bodies, which suggested an exogenous source (Fig. 1a, b).

## Discussion

Acquired NLDO is one of the most common causes of chronic epiphora [2, 9]. In older populations, eyelid malpositioning and skin laxity are common culprits. It is prudent to check for punctal apposition, lagophthalmos, and Bell phenomenon. In younger adults, true NLDO predominates and affects females more than males (65–73%) [9]. The differential for a duct obstruction includes chronic inflammation secondary to conditions such as dacryocystitis or conjunctivitis, vascular abnormalities such as granulomatosis with polyangiitis, or mechanical causes, like blockage from primary lacrimal system tumors and naso-orbital-ethmoid fractures.

Most reports of ocular complaints secondary to cosmetics are immediate after application and likely due to contact dermatitis or allergic response [8]. Chronic reactions are relatively poorly reported on and often reference patients over 40 [10]. There have been only a few reports of pigmented granules, often believed to be foreign particles from cosmetic products, within the lacrimal sac and conjunctiva of patients with NLDO [8]. A summary of the case reports from the last 10 years are listed in Table 1 [5, 10–12]. However, whether these granules were the cause of duct obstruction by virtue of being a nidus for chronic inflammation is unclear [1]. In these reports, histopathological examination showed particles in stromal cells, macrophages, or within a dacryolith that caused canalicular obstruction [4, 5, 7], suggesting these particles were relatively immunologically inert. Another case noted a non-granulomatous reaction to pigmented material with symptoms over a shorter time frame [10]. Here, we show a rare case of birefringent material, possibly content from a cosmetic product, associated with classical foreign body granulomatous reaction leading to insidious occlusion of the nasolacrimal duct and this patient’s persistent epiphora.

**Table 1:** Summary of case reports on cosmetic-associated nasolacrimal duct obstruction from last 10 years

Authors	Year	Age, sex	Clinical description
Clifford et al. [5]	2011	69, F	Presented with epiphora and NLDO. Underwent left external DCR and found to have abnormal pigmentation at the entrance to the common nasolacrimal sac. Histopathology showed fibrous tissue with brown pigment and birefringent material
Gupta et al [11]	2019	62, F	Presented with bilateral epiphora. Found to have abnormally pigmented canaliculi during DCR. Histopathology showed fibrotic changes with abundant stromal pigmentation
Scollo et al [10]	2021	35, F	Presented with acute, left dacryocystitis and underwent external DCR. Gross morphology was non-revealing. Histopathology showed signs of chronic inflammation, brown pigment, and polarizable elements in the lacrimal sac mucosa
Ali et al [12]	2022	51, F	Presented with partial left NLDO and underwent balloon dacryoplasty. During endoscopic visualization of the inferior NLD meatus opening, dark pigmented streaks were grossly seen. Patient was a consistent Kohl eyeliner user

This case highlights the importance of eliciting a comprehensive history, including patterns of cosmetics and other exogenous material use, when evaluating a patient with persistent ocular complaints. Pigmented granules can be seen in a wide variety of pathologies, ranging from innate, benign melanosis to melanoma. However, the patient noted she used tubing mascara daily for over 15 years, which provided key historical context for interpreting her histopathological results. This information enabled us to differentiate her results from more concerning pathologies, and reduced the need for further, invasive work-up.

While prior reports either had conjunctival or punctal pigmentation [4], or initial obstruction on lacrimal syringing [10], we recognize the added complexity in diagnosis in that this patient’s presentation and history was consistent with dry eye disease. Given the findings in this case, a high degree of suspicion should be held for NLDO in chronic makeup users when there is no clinical improvement in tearing with dry eye or allergic conjunctivitis treatment. Ophthalmologists should be aware that cosmetic-grade products can lead to a chronic inflammatory response and/or structural abnormalities within the nasolacrimal duct system, and that this pathology can co-present with physical exam findings consistent with dry eye and allergic conjunctivitis. Given how widespread makeup usage is, eyelid hygiene should be stressed in all users, even in those without an overt allergic or inflammatory reaction to their cosmetic products. Early consideration of this rare cause of NLDO can assist in patient guidance; makeup discontinuation should be suggested at the first signs of epiphora to help prevent total obstruction.

Evaluating epiphora can be difficult and requires a broad differential and open communication with the patient. Here we share a case that illustrates that inflammation due to cosmetic or other exogenous products, even without irritating symptoms, should be considered in this differential and used to inform initial history and physical evaluation tactics.
